# Optimising breathlessness triggered services for older people with advanced diseases: a multicentre economic study (OPTBreathe)

**DOI:** 10.1136/thoraxjnl-2021-218251

**Published:** 2022-08-15

**Authors:** Deokhee Yi, Charles C Reilly, Gao Wei, Irene J Higginson, OPTBreathe collaborators, Noel Baxter

**Affiliations:** 1 Cicely Saunders Institute of Palliative Care, Policy and Rehabilitation, King's College London, London, UK; 2 Physiotherapy, King's College Hospital NHS Foundation Trust, London, UK; 3 Palliative care, King's College Hospital NHS Foundation Trust, London, UK; 4 King's College Hospital NHS Foundation Trust, London, UK

**Keywords:** Lung Cancer, Health Economist, Palliative Care

## Abstract

**Background:**

In advanced disease, breathlessness becomes severe, increasing health services use. Breathlessness triggered services demonstrate effectiveness in trials and meta-analyses but lack health economic assessment.

**Methods:**

Our economic study included a discrete choice experiment (DCE), followed by a cost-effectiveness analysis modelling. The DCE comprised face-to-face interviews with older patients with chronic breathlessness and their carers across nine UK centres. Conditional logistic regression analysis of DCE data determined the preferences (or not, indicated by negative β coefficients) for service attributes. Economic modelling estimated the costs and quality-adjusted life years (QALYs) over 5 years.

**Findings:**

The DCE recruited 190 patients and 68 carers. Offering breathlessness services in person from general practitioner (GP) surgeries was not preferred (β=−0.30, 95% CI −0.40 to −0.21); hospital outpatient clinics (0.16, 0.06 to 0.25) or via home visits (0.15, 0.06 to 0.24) were preferred. Inperson services with comprehensive treatment review (0.15, 0.07 to 0.21) and holistic support (0.19, 0.07 to 0.31) were preferred to those without. Cost-effectiveness analysis found the most and the least preferred models of breathlessness services were cost-effective compared with usual care. The most preferred service had £5719 lower costs (95% CI −6043 to 5395), with 0.004 (95% CI −0.003 to 0.011) QALY benefits per patient. Uptake was higher when attributes were tailored to individual preferences (86% vs 40%).

**Conclusion:**

Breathlessness services are cost-effective compared with usual care for health and social care, giving cost savings and better quality of life. Uptake of breathlessness services is higher when service attributes are individually tailored.

**WHAT IS ALREADY KNOWN ON THIS TOPIC**

- Holistic short-term multiprofessional breathlessness triggered services reduce distress and the psychological outcomes of anxiety and depression in patients with chronic breathlessness.

**WHAT THIS STUDY ADDS**

- We propose the most cost-effective models of breathlessness services by identifying attributes to prioritise, especially with limited resources, and by conducting Markov modelling.

**HOW THIS STUDY MIGHT AFFECT RESEARCH, PRACTICE OR POLICY**

- Breathlessness services favoured by patients and carers are in accord with clinician recommendations for comprehensive treatment review, a holistic approach to manage breathlessness and a flexible individual approach.
- Breathlessness services offer cost savings in healthcare of more than £50 000 for every quality-adjusted life year gained.

## Introduction

Breathlessness is common in respiratory diseases and many other conditions.^1^ In the advanced stages of illness, breathlessness often becomes severe, debilitating and frightening, resulting in emergency visits and hospitalisations.^2–5^ When breathlessness continues despite optimal treatment of the underlying condition, it is often referred to as chronic or refractory.^6 7^ People with such breathlessness often have multiple symptoms, which increase as their disease progresses. Breathlessness results in high costs to health and social care systems. It affects family members and those close to the patients (hereafter called carers), with high informal care costs that increase overall societal costs by >250%.^8^

Consequently, a holistic approach involving early palliative care is often proposed as beneficial in advanced respiratory disease.^9^ A meta-analysis including 37 articles trialling 18 services found that holistic short-term multiprofessional breathlessness triggered services (BSs) reduce distress in patients with chronic breathlessness due to advanced diseases and the psychological outcomes of anxiety and depression.^10^ These services, such as the London-based BS and those in Australia, New Zealand and Germany, combine expert respiratory, palliative and therapy (physiotherapy and occupational therapy) assessment and review, plus a toolkit of evidence-based, non-pharmacological and pharmacological interventions.^7 11–16^ However, a Delphi exercise found divergent expert opinions about how services might be best provided, especially whether these should be from general practitioner (GP) surgeries, outpatient clinics or directly to people’s homes.^9^ There is no evidence of which service attributes to prioritise, especially with limited resources. Cost-effectiveness data to inform decision-making are lacking. Therefore, the study to optimise cost-effective support for older patients with refractory breathlessness (OPTBreathe) aimed to identify what attributes of BSs patients and their carers value most. We then aimed to conduct an economic evaluation of BSs in order to propose the most cost-effective models of BSs based on clinical effectiveness and available resources.

## Methods

This economic study had two components: a discrete choice experiment (DCE)^17–19^ to determine the attributes influencing preferences for BSs, followed by a cost-effectiveness Markov modelling.

DCE is a quantitative method increasingly used by health economists and in healthcare to elicit participants’ (patients, payers, carers) relative preference weights for service attributes that will affect their use, without directly asking them to state their preferred options. In a DCE, participants are presented with alternative hypothetical scenarios containing different attributes. Participants make a choice between competing scenarios, each of which consists of a combination of the studied attributes. Using the derived parameters from the DCE results, combined with epidemiological data and existing trial data, Markov modelling^20^ (online supplemental figure S2) was conducted to determine cost-effectiveness.^6 21 22^ Markov modelling is an analytical framework frequently used in economic evaluation of healthcare interventions. Reporting follows the Consolidated Health Economic Evaluation Reporting Standards,^23^ Conjoint analysis checklist^24^ and the guidelines for decision analytic modelling^25^ (see the Ethics approval section).

10.1136/thoraxjnl-2021-218251.supp1 Supplementary data

### DCE to elicit preference and acceptability

The DCE sought to determine (1) the absolute and relative importance of attributes, (2) the willingness to wait for attributes, and (3) the probabilities of uptake (ie, level of acceptance) of varying packages of BSs.

#### Definition of attributes

**Figure 1:**
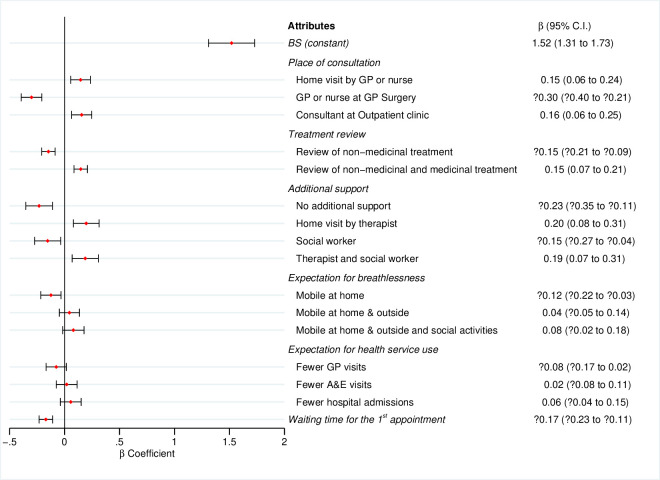
Preferences for attributes and levels of breathlessness service: results of the regression analysis from all patients and carers. The sign and magnitude of coefficients indicate the direction and strength of preferences for each attribute. Positive coefficients represent the degree of preference for the corresponding attribute and negative coefficients the degree of ‘negative preference’ or rejection of the corresponding attribute. A&E, accident and emergency; BS, breathlessness service; GP, general practitioner.

Attributes were built on findings from literature reviews, service modelling, consultation with this study’s collaborators and patient experts, and data from the trial of BS,^11^ including qualitative interviews with patients.^12 13 15^ Preliminary secondary analysis of these data and review by our patient and public involvement and engagement (PPIE) group suggested that the key attributes should be grouped according to place of consultation, nature of treatment review, additional support offered, expectation for change in breathlessness and expectation for change in healthcare use (see figure 1). The PPIE input revised the descriptive words used. We prepared hypothetical choices, with six sets per participant, for the DCE (see online supplemental figure S1 for details).

#### DCE settings

The study was opened in nine participating study sites across the UK, with a mix of urban and more rural areas (online supplemental table S1), including respiratory medicine and lung cancer clinics, palliative care services, from patients admitted to hospital for breathlessness, physiotherapy services, and integrated respiratory teams.

#### DCE participants

We recruited consenting patients who were 65 or older, with a medical diagnosis of COPD, lung cancer or interstitial lung disease (ILD), and with breathlessness refractory to optimal medical management in the view of the treating clinician, with a severity level of 2, 3, 4 or 5 on the Medical Research Council Dyspnoea Scale. Sample size calculation is described in online supplemental file 1.

We also recruited a consenting family member/friend/lay carer who was designated as the closest family member or caregiver to the participating patient. Patients with breathlessness of unknown cause, with chronic hyperventilation syndrome, or without capacity to fully comprehend and retain information about the study or those unable to engage in an interview were excluded.

All interviews were conducted face-to-face using a standard questionnaire specifically designed for the project, asking for experiences in managing breathlessness, questions about service use, and clinical and sociodemographic information, in addition to six choice questions of the DCE, as well as open questions for explanation (reasons for choice) and expansion (other comments). Details of administration of the DCE questionnaires and data collection are found in online supplemental file 1.

#### Analysis of choice question data

We described the characteristics of patients and carers and examined if anyone chose ‘neither’ in all six choices. We used a conditional logit model with individual fixed effects, considering the independence of irrelevant alternative, random taste variation (each respondent has different coefficients) and multiple observations from the same respondent. The magnitude and sign (+/−) of the coefficients on the attributes and the levels represent the strength and direction of preferences. The strength of preference can be inferred from the magnitude of the coefficients and the order of preference for attributes or levels can be established. After obtaining coefficients on the attributes and their groups, representing the relative weights in utility from the BS, we calculated the probability of acceptance of the proposed BS, to be used in the Markov model. We also calculated the CI for probabilities using bootstrapping.

Subgroup analyses were conducted by patients and carers, London-based and non-London-based, and gender of the patients. All analyses were conducted using STATA V.16.

#### Analysis of text from open-ended questions

To explore the reasons why patients and carers chose certain options, free-text answers to the questions, asking respondents to make comments or suggestions regarding services to manage breathlessness, were analysed using a thematic analysis approach.

### Economic modelling for cost-effectiveness

We developed a Markov model to simulate the effect of BS in a cohort of patients and to estimate the cost-effectiveness of different models of BS compared with usual care. We populated the model with data from the breathlessness trial (of a holistic multiprofessional service where patients with severe continuing breathlessness were referred)^6^ and published mortality rates (see online supplemental tables S2 and S3). The analysis was conducted from a national health service perspective.

#### Development of model structure

We defined a starting cohort by sample characteristics (eg, 75-year-old men with COPD), reflecting the epidemiology of a disease. The transition states in the Markov model were defined mutually exclusive (eg, receiving usual care, death). Intervention (taking up BS plus usual care) and associated states (eg, stable with improvement, stable with extended effects, no change) were also defined. Cycle length was set as 12 weeks (or 3 months) and we used 20 cycles (or 5 years) for time horizon to capture the clinical problems and intervention effects. We assumed that the effects of BS disappear after 12 weeks (first cycle) in case of the BS plus and usual care. For BS with a lasting effect scenario, we assumed that the effects of BS lasted another 12 weeks and disappear after the second cycle. We used age-specific and sex-specific all-cause mortality in the initial stage and used age-specific and sex-specific respiratory mortality (online supplemental table S3) in later stages. The yearly discount rate of 3.5% was applied considering a cycle length of 0.25 years.

#### Incremental costs per quality-adjusted life years

We used the EuroQol-5 Dimension (EQ-5D) index scores and health and social care costs in the BS plus usual care group and the usual care only group before and after a BS in the trial data. Using Monte Carlo simulation with 1000 in the defined cohort, we estimated the incremental cost-effectiveness ratio (ICER), which is defined as: (cost of intervention−cost of control) divided by (effect of intervention−effect of control).

#### Integration of results from the DCE

The probability of acceptance was calculated for the most acceptable BS, and the least acceptable BS, and used in the Markov model to examine changes in the cost-effectiveness of the BS with given configurations.

#### Sensitivity analysis

To assess the robustness of the results to the realistic variations in the level of underlying data, we conducted a sensitivity analysis. We estimated the model for a different cohort (75-year-old women with COPD) and for two different configurations of BS as deterministic sensitivity analysis. CIs around costs and quality-adjusted life years (QALYs) were calculated using delta methods (see online supplemental table S5). For uncertainty around the ICER, the cost-effectiveness acceptability curve (CEAC) against willingness to pay was explored. We used Treeage Pro Software V.2020 for this modelling.

## Results

### Discrete choice experiment

We recruited 190 patients and 68 carers: 130 patients had COPD, 18 had ILD and 40 had lung cancer (with 2 missing) (table 1). Patients had a mean age of 75 years, 35% were women and 30% were living alone. Of the patients, 21% were widowed and 62% were married/partnered, and 85% were living comfortably or coping on present income. Carers were on average 64 years old and 86% were women. Two-thirds of the carers were spouse/partner and 21% were daughter/son, and 90% had a religious faith. In this study, 49% of the patients and 66% of the carers were recruited from hospitals in the London area.

**Table 1:** Characteristics of patients and carers who participated in the survey

	Patients (n=190)	Carers (n=68)
Diagnosis*		
COPD	130 (69)	32 (47)
Interstitial lung disease	18 (10)	14 (30)
Lung cancer	40 (21)	22 (33)
Age†	74.9 (6.1)	64.0 (13.5)
Male/female†	124 (65)/66 (35)	9 (14)/55 (86)
Ethnicity		
White	180 (95)	61 (90)
Non-white	8 (4)	7 (10)
Marital status		
Single	15 (8)	8 (12)
Widowed	39 (21)	
Married/partnership	118 (62)	59 (87)
Divorced/separate	18 (9)	1 (1)
Financial status†		
Living comfortably on present income	77 (41)	30 (45)
Coping on present income	86 (45)	23 (34)
Difficult on present income	10 (5)	8 (12)
Very difficult on present income	2 (1)	2 (3)
Do not know	2 (1)	
Refusal/prefer not to say	6 (7)	4 (6)
Educational attainment*		
Left school at 15 years old or under	119 (63)	26 (38)
Left school at 16–17 years old	45 (24)	19 (28)
Left school at 18–19 years old	5 (3)	4 (6)
Postsecondary/vocational qualifications	8 (4)	8 (12)
University	12 (6)	11 (16)
Religious faith (=1 if yes)†	97 (51)	35 (52)
Living alone (=1 if yes)	57 (30)	0 (0)
Study site		
London	93 (49)	45 (66)
Other areas of England	97 (51)	23 (34)
Relationship to patient†		
Spouse/partner		46 (68)
Son/daughter		14 (21)
Parent		2 (3)
Friend		1 (1)
Others		2 (3)

Mean (SD) for age; n (%) for the rest.

*Two missing in diagnosis and one in educational attainment in the patient group.

†Two missing in age, four in female, one in financial status, one in religious faith and three in relationship to the patient in the carer group.

In the final analysis, 1535 choice questions or 4605 observations from 256 participants were used. The frequencies of each attribute (online supplemental table S4) did not show the main drivers of choices.

### Importance of attributes of BS

Respondents had a strong preference for any combinations of BS (β=1.52, 95% CI 1.31 to 1.73; figure 1), indicating that respondents always wanted BS. BS offered at GP surgeries was not preferred (showing a negative preference; β=−0.30, 95% CI –0.40 to −0.21). In contrast offering BS was preferred at hospital outpatient clinics (β=0.16, 95% CI 0.06 to 0.25) or via home visits (β=0.15, 95% CI 0.06 to 0.24). BS with comprehensive treatment review (β=0.15, 95% CI 0.07 to 0.21) and holistic support (β=0.19, 95% CI 0.07 to 0.31) was preferred to usual services. Respondents did not prefer BS with no additional support (β=−0.23, 95% CI –0.35 to −0.11) or with social workers alone (β=−0.15, 95% CI –0.27 to −0.04).

### Probability of taking up various BS

**Figure 2:**
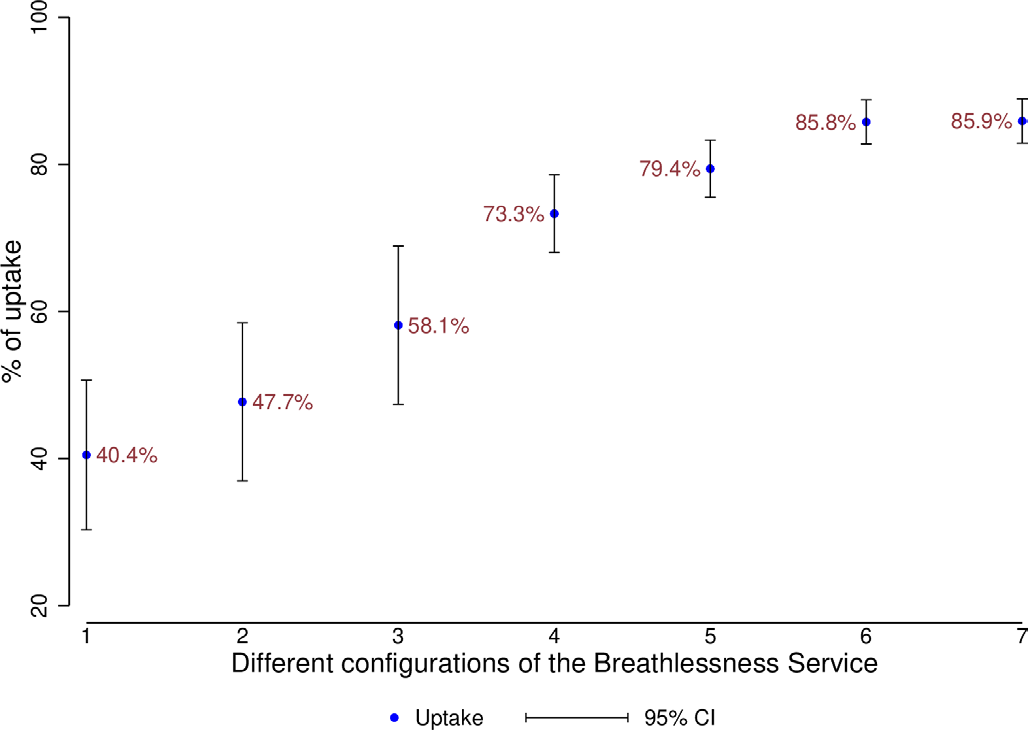
Probabilities of taking up breathlessness triggered services (BSs) with different configurations of attributes. Two attributes were fixed: expectation for breathlessness as ‘being more mobile at home & outside and enjoying social activities’ and expectation for health service as ‘having fewer hospital admissions’. Example of BS: (1) general practitioner (GP) surgery, review of non-medicinal treatment only, no additional support and waiting for 8 weeks to have the first appointment; (2) GP surgery, review of non-medicinal treatment and medicinal treatment, no additional support, and waiting for 8 weeks to have the first appointment; (3) GP surgery, review of non-medicinal treatment and medicinal treatment, support from therapists and social workers, and waiting for 8 weeks to have the first appointment; (4) GP surgery, review of non-medicinal treatment and medicinal treatment, support from therapists and social workers, and waiting for 4 weeks to have the first appointment; (5) GP surgery, review of non-medicinal treatment and medicinal treatment, support from therapists and social workers, and waiting for 2 weeks to have the first appointment; (6) home visit by a GP or a nurse, review of non-medicinal treatment and medicinal treatment, support from therapists and social workers, and waiting for 2 weeks to have the first appointment; and (7) visit to outpatient clinic, review of non-medicinal treatment and medicinal treatment, support from therapists and social workers, and waiting for 2 weeks to have the first appointment.

The least valued BS was determined, according to the estimated coefficients, as the services offered at GP surgeries, reviewing non-medicinal treatment, no additional support and 8 weeks of waiting time for the first appointment. The probability of taking up such BS was 40.4% (1 in figure 2). As patients and carers valued review of medicinal (pharmacotherapy) treatment, support from therapists and social workers, and shorter waiting time, the probability of taking up BS increased from 1 to 5 incrementally (figure 2). For example, when reviewing medicinal treatment was added to the BS, the probability of uptake increased to 47.7%. The most preferred configuration of the BS was where patients visit outpatient clinics to see consultants, review of both medicinal and non-medicinal treatment, support by a physiotherapist, an occupational therapist and a social worker, and with 2 weeks to wait to get the first appointment (figure 2). It needs to be noted that BS configuration 6, offering home visits instead of appointments at outpatient clinics, had a probability of uptake as high as BS configuration 7 (figure 2). Patients and carers preferred to see consultants at outpatient clinics, but were willing to choose to take part in BS when home visits were provided.

### Subgroup analysis

Carers had preferences different from patients (log likelihood ratio test: X^2^=21.77, p<0.04; see online supplemental table S6). Carers stated a stronger preference for BS with home visits from GPs, both medicinal and non-medicinal review, and involvement from social workers and therapists, than were patients. Overall preferences of female patients differed from those of male patients (log likelihood ratio test: X^2^=797.69, p<0.001; see online supplemental table S7). Women preferred home visits and social workers along with therapists, while men preferred outpatient clinics and therapists only. There was no overall difference in recruitment site (London and other areas of England) (log likelihood ratio test: X^2^=8.58, p<0.73; see online supplemental table S8).

We defined the base cases in modelling using these findings (difference between female and male patients and no difference between London and other areas of England). In other words, the take-up of BS in the ‘male 75’ group was derived with estimated results from male patients at all sites.

### Analysis of free-text comments

Sixty-four participants provided free-text comments and 158 made suggestions for future BSs. Regarding the reasons for preferring hospital outpatient clinics or home visits to GP surgeries, respondents felt that GPs could not provide specialist care for breathlessness and said they try to avoid visiting GP due to a perceived risk of infection. Eleven patients wanted to have all the treatments and services at home.

### Markov model estimation

#### Cost-effectiveness from deterministic analysis

We built a Markov model based on three alternatives: status quo (usual care), BS and BS with extended effects (the 3rd branch in the online supplemental figure S3). When the Markov model was estimated for a 75-year-old man over 5 years, the difference in costs between usual care only (no BS) and BS plus usual care was –£663 (95% CI −1076 to −250), and that between usual care only and BS with extended effects plus usual care it was –£5086 (95% CI −5469 to −4703) (table 2). The difference in QALYs was 0.013 (95% CI 0.004 to 0.022) in both cases. For women, the difference in costs was –£749 (95% CI −1100 to −398) and –£5719 (95% CI −6043 to 5395), and the difference in QALYs was 0.013 (95% CI 0.005 to 0.021) and 0.004 (95% CI −0.003 to 0.011), respectively (table 2). As changes in costs were negative and changes in QALYs were positive, providing BS to 75-year-old adults for 12 weeks was cheaper than usual care and the quality of life improved when considering the uptake chances of this population. Negative ICERs with lower costs and better outcomes in BS imply that offering BS to 75-year-old adults is cost-effective (see online supplemental table S5 for details).

**Table 2:** Cost-effectiveness of the breathlessness service (BS) for 5 years: results of the Markov model estimation

	Usual care onlyvs usual care plus BS	Usual care only vs usual care plus BS with lasting effects
75-year-old man, p(uptake)=0.85		
Costs (£)	−663 (−1076 to −250)	−5086 (−5469 to −4703)
Health outcomes (QALY)	0.013 (0.004 to 0.022)	0.013 (0.004 to 0.022)
ICER (£/QALY)	−50 789	−389 776
75-year-old woman, p(uptake)=0.87		
Costs (£)	−749 (−1100 to −398)	−5719 (−6043 to 5395)
Health outcomes (QALY)	0.013 (0.005 to 0.021)	0.004 (−0.003 to 0.011)
ICER (£/QALY)	−56 242	−1 454 683

95% CIs are in parentheses.

BS is designed to involve consultations with a specialist at an outpatient clinic, review of both medicinal and non-medicinal treatments, home visits by therapists, and support from a social worker.

Costs were calculated based on the National Health Service (NHS) reference costs, and unit costs of health and social careby the Personal Social Services Research Unit (PSSRU).

Better mobility and independence at home and outside home and more social activities are anticipated. Fewer hospital admissions are expected and patients need to wait 2 weeks to get the first appointment.

ICER=(costs_2_−costs_1_)/(QALY_2_−QALY_1_), where 1 and 2 represent alternatives compared. Costs are in 2014 British pound sterlling.

ICER, incremental cost-effectiveness ratio; QALY, quality-adjusted life years.

When the CEACs were explored for each scenario to examine the uncertainty around the ICER, the acceptability was 1 (complete acceptance) in the ranges of willingness to pay.

## Discussion

This multicentre study is the first to provide a comprehensive economic assessment of BSs for advanced diseases. It couples patient and carer preferences with cost-effectiveness modelling to determine optimal configurations for BSs. Patients and carers valued any configurations of BS over usual care. They preferred BS offering consultations at outpatient clinics or by home visits rather than at GP surgeries and wanted review of both medicinal (pharmacotherapy) and non-medicinal treatments and holistic support from therapists and social workers. The Markov model estimated the cost-effectiveness beyond the trial period (12 weeks) for up to 5 years and found that BS was cost-effective and could contribute to savings in health and social care systems.

COPD accounts for £800 million in direct healthcare costs in the UK.^26^ The indirect costs of COPD are substantial (24 million lost working days per annum).^27^

Costs rise in advanced diseases.^28^ Therefore, finding cost-effective solutions is essential. There is good evidence regarding pulmonary rehabilitation in earlier stages of disease.^29^ However, uptake of pulmonary rehabilitation varies, especially in advanced diseases where patients find it difficult to leave home. Our results indicate that in the advanced stages of disease BSs provide cost savings and better quality of life, even in instances of lower uptake at only ~40%. When the uptake is higher, savings and quality of life improvements are greater. BSs are designed to be a brief intervention, with usually around three indepth contacts, with a longer lasting impact due to the focus on continuing patient self-management and empowerment.^9 11 12^

We were surprised to find that patients and carers preferred BS at outpatient clinics or via home visits and not at GP surgeries. Free-text answers supported this finding: patients and carers thought that GPs were not the experts in breathlessness management and that visiting GP surgeries might risk contracting infections. This finding is at odds with a Delphi exercise with clinicians which suggested that BSs should be integrated into existing GP services.^30^ However, our DCE findings based on patient preferences are in accord with clinician recommendations for a comprehensive treatment review, a holistic approach to manage breathlessness and a flexible individual approach. The risk raised by patients of infection in GP surgeries and possibly hospitals is brought clearly into focus by the COVID-19 pandemic. BSs offered online or remotely, or from treatment hubs, might be a good alternative. These could reduce the need for travel and allow a combined expert and GP review. Efficient community services providing virtual at-home services with shorter waiting time could encourage patients to take up BS and be a future model. Our results also highlight the importance of responding to patient preferences, with estimated uptake doubling (from 40% to 86%) when the most preferred type of service was offered.

We assessed the cost-effectiveness of BSs beyond the time horizon used in the trials, conducting cost-effectiveness analysis with a Markov model. This helps commissioners and healthcare professionals decide to adopt BSs in their communities. We used the actual views from patients and carers in the decision process in the cost-effectiveness analysis by incorporating the uptake rates of the BSs calculated from the DCE data. Unlike the assumptions or scenarios usually used in this type of analysis, we calculated the cost-effectiveness of BSs based on the stated preferences of patients and carers, which can be realistic.

Our study has several limitations. DCEs suffer from hypothetical bias; our respondents may not have selected what they would do in reality. We assuaged this bias by including only older individuals with relevant illnesses and moderate to severe breathlessness, who therefore had experience of the relevant services and health problems and so were better able to judge the situation. We also explored the reasons for choices in open comments. This enriched the data and suggests ways in which some negative preferences, such as for GP surgeries, may be overcome, for example by reducing the risk of infection in waiting rooms and providing expert support at GP surgeries. We included both patients and their carers; carers may play an important role in influencing patient preferences and choices.^31^ We found some differences in preferences between patients and carers regarding BS composition. Neither preferred GP surgeries; however, compared with patients, carers had a slightly stronger preference for home visits and social worker involvement. The preference for social worker involvement may reflect carer-specific supportive needs.

We need to consider how our sample represents the population of interest. We identified patients via nine centres across England: South East, Midlands, East and North East, as well as London. When compared with the national audit data,^32^ our sample was slightly older (75 years vs 71 years) because we intendedly approached older adults, being of specific interest and often less able to attend pulmonary rehabilitation and similar services. In our sample, 49% of patients and 66% of carers were recruited from London. Other patients were more often recruited from hospital centres from urban UK populations. According to population statistics, only 17% of the UK live in rural areas and 68% of the world population will live in cities by 2050. Therefore, our data are highly relevant to most people affected by severe breathlessness. Our results may not be generalised to rural areas; further research would need to investigate this. We found no differences between London and other areas of England, a finding supported by the similar referral rates for pulmonary rehabilitation (20% in London and 19% in the rest of England in the UK quality indicator data).^33^

Cognitive burden can be a limitation when conducting DCEs, especially among older adults with chronic or advanced diseases. We determined the number of choice questions and attributes by detailed piloting. We trained research nurses, emphasising the risks of cognitive burden and offering rests, etc. One indicator of burden is poor question completion rates. We obtained rich data, with only one choice question unanswered. Therefore, we found that DCEs can be used for older adults with advanced diseases. These produced important information regarding service development and planning, which could inform future research methods.

In the modelling, we used a 5-year time horizon, although survival of patients with advanced diseases (eg, ILD, lung cancer) could vary and in instances would be shorter. We modelled different time periods of the effect of BS, including only 12 weeks. When we assumed that the effects of BS lasted longer (than 12 weeks), the cost savings were larger. BSs warrants future research with longer follow-up periods, which examines the effects of boosts of BSs such as a follow-up or refresher consultation, including mortality. We did not include social care costs, informal care costs or costs from lost productivity. Caution is needed regarding the shift of costs between different care settings (eg, hospital, community, home) or from formal to informal care. However, the BS trial did not find an increase in informal care costs in the intervention arm,^6^ and if anything our cost savings are likely underestimated.

Data were collected before the COVID-19 pandemic, which has changed the values and attitudes towards services and the risk of infection. However, our data can be considered in the light of these changing attitudes and can help shape breathlessness services in COVID-19 pandemic situations to better help support patients with long-lasting breathlessness and their carers.

## Conclusion

Patients with chronic breathlessness and their carers valued and accepted BSs, which were cost-effective, offering lower costs and better quality of life (savings of £50 000 per patient, plus additional QALY) compared with usual care. The uptake and cost-effectiveness of BSs are higher when services are tailored to individual preferences, with uptake increasing from 40% to 86%.

## Data Availability

Data are available upon reasonable request. We can share the protocol, consent form, analysis plan, training and other relevant DCE materials. Data from the survey are approved for use by the research team and appropriately qualified researchers trained and supervised by them. Fully anonymised data with other studies for secondary analysis could be considered. De-identified participant data will be made available to bona fide researchers registered with an appropriate institution within 3 months of publication. However, the research team will need to be satisfied that any proposed publication is of high quality, honours the commitments made to the study participants in the consent documentation and ethical approvals, and is compliant with relevant legal and regulatory requirements (eg, relating to data protection and privacy). The research team will have the right to review and comment on any draft manuscripts before publication. Data will be made available in line with the policy and procedures of the Cicely Saunders Institute of Palliative Care, Policy and Rehabilitation. Those wishing to request access should email deok_hee.yi@kcl.ac.uk.

## References

[1] Booth S , Bausewein C , Higginson I , et al . Pharmacological treatment of refractory breathlessness. Expert Rev Respir Med 2009;3:21–36. 10.1586/17476348.3.1.21 20477280

[2] Gysels MH , Higginson IJ . The lived experience of breathlessness and its implications for care: a qualitative comparison in cancer, COPD, heart failure and MND. BMC Palliat Care 2011;10:15. 10.1186/1472-684X-10-15 22004467 PMC3206451

[3] Malik FA , Gysels M , Higginson IJ . Living with breathlessness: a survey of caregivers of breathless patients with lung cancer or heart failure. Palliat Med 2013;27:647–56. 10.1177/0269216313488812 23703238

[4] Janssen DJA , Spruit MA , Uszko-Lencer NH , et al . Symptoms, comorbidities, and health care in advanced chronic obstructive pulmonary disease or chronic heart failure. J Palliat Med 2011;14:735–43. 10.1089/jpm.2010.0479 21510771

[5] Janssen DJA , Spruit MA , Schols JMGA , et al . A call for high-quality advance care planning in outpatients with severe COPD or chronic heart failure. Chest 2011;139:1081–8. 10.1378/chest.10-1753 20829337

[6] Higginson IJ , Bausewein C , Reilly CC , et al . An integrated palliative and respiratory care service for patients with advanced disease and refractory breathlessness: a randomised controlled trial. Lancet Respir Med 2014;2:979–87. 10.1016/S2213-2600(14)70226-7 25465642

[7] Philip J , Wiseman R , Eastman P , et al . Mapping non-malignant respiratory palliative care services in Australia and New Zealand. Aust Health Rev 2020;44:778–81. 10.1071/AH19206 32943138

[8] Dzingina MD , McCrone P , Higginson IJ . Does the EQ-5D capture the concerns measured by the palliative care outcome scale? mapping the palliative care outcome scale onto the EQ-5D using statistical methods. Palliat Med 2017;31:716–25. 10.1177/0269216317705608 28434392

[9] Maddocks M , Brighton LJ , Farquhar M . Holistic services for people with advanced disease and chronic or refractory breathlessness: a mixed-methods evidence synthesis 2019;7:22. 31241880

[10] Brighton LJ , Miller S , Farquhar M , et al . Holistic services for people with advanced disease and chronic breathlessness: a systematic review and meta-analysis. Thorax 2019;74:270–81. 10.1136/thoraxjnl-2018-211589 30498004 PMC6467249

[11] Higginson IJ , Bausewein C , Reilly CC , et al . An integrated palliative and respiratory care service for patients with advanced disease and refractory breathlessness: a randomised controlled trial. Lancet Respir Med 2014;2:979–87. 10.1016/S2213-2600(14)70226-7 25465642

[12] Reilly CC , Bausewein C , Pannell C , et al . Patients' experiences of a new integrated breathlessness support service for patients with refractory breathlessness: results of a postal survey. Palliat Med 2016;30:313–22. 10.1177/0269216315600103 26311570 PMC4778380

[13] Gysels M , Reilly CC , Jolley CJ , et al . How does a new breathlessness support service affect patients? Eur Respir J 2015;46:1515–8. 10.1183/13993003.00751-2015 26381516

[14] Schunk M , Le L , Syunyaeva Z , et al . Effectiveness of a specialised breathlessness service for patients with advanced disease in Germany: a pragmatic fast-track randomised controlled trial (BreathEase). Eur Respir J 2021;58:2002139. 10.1183/13993003.02139-2020 33509957

[15] Gysels M , Reilly CC , Jolley CJ , et al . Dignity through integrated symptom management: lessons from the breathlessness support service. J Pain Symptom Manage 2016;52:515–24. 10.1016/j.jpainsymman.2016.04.010 27650009

[16] Dorman S , Jolley C , Abernethy A , et al . Researching breathlessness in palliative care: consensus statement of the National cancer research Institute palliative care breathlessness subgroup. Palliat Med 2009;23:213–27. 10.1177/0269216309102520 19251835

[17] Clark MD , Determann D , Petrou S , et al . Discrete choice experiments in health economics: a review of the literature. Pharmacoeconomics 2014;32:883–902. 10.1007/s40273-014-0170-x 25005924

[18] Lancsar E , Fiebig DG , Hole AR . Discrete choice experiments: a guide to model specification, estimation and software. Pharmacoeconomics 2017;35:697–716. 10.1007/s40273-017-0506-4 28374325

[19] Lancsar E , Louviere J . Conducting discrete choice experiments to inform healthcare decision making: a user's guide. Pharmacoeconomics 2008;26:661–77. 10.2165/00019053-200826080-00004 18620460

[20] Rudmik L , Drummond M . Health economic evaluation: important principles and methodology. Laryngoscope 2013;123:1341–7. 10.1002/lary.23943 23483522

[21] Farquhar MC , Prevost AT , McCrone P , et al . The clinical and cost effectiveness of a breathlessness intervention service for patients with advanced non-malignant disease and their informal carers: mixed findings of a mixed method randomised controlled trial. Trials 2016;17:185. 10.1186/s13063-016-1304-6 27044249 PMC4820876

[22] Farquhar MC , Prevost AT , McCrone P , et al . Is a specialist breathlessness service more effective and cost-effective for patients with advanced cancer and their carers than standard care? findings of a mixed-method randomised controlled trial. BMC Med 2014;12:194. 10.1186/s12916-014-0194-2 25358424 PMC4222435

[23] Husereau D , Drummond M , Petrou S , et al . Consolidated Health Economic Evaluation Reporting Standards (CHEERS)--explanation and elaboration: a report of the ISPOR Health Economic Evaluation Publication Guidelines Good Reporting Practices Task Force. Value Health 2013;16:231–50. 10.1016/j.jval.2013.02.002 23538175

[24] Bridges JFP , Hauber AB , Marshall D , et al . Conjoint analysis applications in health--a checklist: a report of the ISPOR Good Research Practices for Conjoint Analysis Task Force. Value Health 2011;14:403–13. 10.1016/j.jval.2010.11.013 21669364

[25] Philips Z , Ginnelly L , Sculpher M , et al . Review of guidelines for good practice in decision-analytic modelling in health technology assessment. Health Technol Assess 2004;8:1–158. 10.3310/hta8360 15361314

[26] Deaprtment of Health . On the state of the public health: annual report of the chief medical officer 2004, 2005.

[27] Healthcare Commission . Clearing the air: a national study of chronic obstructive pulmonary disease, 2006.

[28] Dzingina MD , Reilly CC , Bausewein C , et al . Variations in the cost of formal and informal health care for patients with advanced chronic disease and refractory breathlessness: a cross-sectional secondary analysis. Palliat Med 2017;31:369–77. 10.1177/0269216317690994 28190370 PMC5405827

[29] McCarthy B , Casey D , Devane D . Pulmonary rehabilitation for chronic obstructive pulmonary disease. Cochrane Database Syst Rev 2015;2:Cd003793. 10.1002/14651858.CD003793.pub3 PMC10008021 25705944

[30] Brighton LJ , Tunnard I , Farquhar M , et al . Recommendations for services for people living with chronic breathlessness in advanced disease: results of a transparent expert consultation. Chron Respir Dis 2019;16:147997311881644–8. 10.1177/1479973118816448 PMC6313262 30789022

[31] Etkind SN , Bone AE , Lovell N , et al . Influences on care preferences of older people with advanced illness: a systematic review and thematic synthesis. J Am Geriatr Soc 2018;66:1031–9. 10.1111/jgs.15272 29512147 PMC6001783

[32] Singh SLS , Andrews R , Garnavos N . National asthma and chronic obstructive pulmonary disease audit programme (NACAP). pulmonary rehabilitation audit report 2019. In: Combined clinical and organisational audit of pulmonary rehabilitation services in England. Scotland and Wales, London, 2020.

[33] The NHS Digital . (the percentage of people with Chronic Obstructive Pulmonary Disease (COPD) and Medical Research Council (MRC) Dyspnoea Scale >=3, 2020. Available: https://digital.nhs.uk/data-and-information/publications/statistical/ccg-outcomes-indicator-set/march-2020 [Accessed 25 Mar 2022].

