# Supplementary material for: Optimising breathlessness triggered services for older people with advanced diseases: a multicentre economic study (OPTBreathe)
Source: Thorax. 2022 Aug 15;78(5):489–95. doi: 10.1136/thoraxjnl-2021-218251 (PMC10176396; doi:10.1136/thoraxjnl-2021-218251)
Supplement: Supplementary data [file thoraxjnl-2021-218251supp001.pdf]

Supplementary information to Yi et al.

Optimising breathlessness triggered services for older people with advanced diseases: a multicentre economic study (OPTBreathe)

Deokhee Yi, Charles C Reilly, Wei Gao, Irene J Higginson on behalf of OPTBreathe collaborators

Contents

Sample size of the discrete choice experiment 1
Design of choice questions 1
Administration of discrete choice experiment questionnaire 1
Table S1. Participating organisations and recruited participants........ 3
Table S2. Parameters in the Markov model in deterministic analysis 4
Table S3. All-cause mortality rate and respiratory mortality rate by age and sex used in the Markov model 5
Table S4. Distribution of levels within attributes among choice questions chosen by participants. 6
Table S5. Incremental cost effectiveness ratio of BSS for 5 years (unit: £)........ 7
Table S6. Preferences for attributes and levels of Breathlessness Service: subgroup analysis by patient and carer 9
Table S7. Preferences for attributes and levels of Breathlessness Service: subgroup analysis by sex of patient 10
Table S8. Preferences for attributes and levels of Breathlessness Service: subgroup analysis by location (London and outside London) 11
Figure S1. Example choice question presented to respondents 12
Figure S2. Decision tree for Markov model analysis of breathless support service........ 13
References 14

Yi D, *et al. Thorax* 2023; 78:489–495. doi: 10.1136/thoraxjnl-2021-218251

Supplementary information to Yi et al.

Sample size of the discrete choice experiment

To calculate the sample size of the DCE, we have considered the following:

1) Regression analysis needs a sample size larger than the number of independent variables. We will be able to enter up to 20 independent variables and retain up to 14, if the rules of thumb suggested by Altman apply (n/10 variables and square root of sample size respectively).[1] For each predetermined subgroup of the main sample (i.e., diagnosis group), a sample size should be larger than 30
2) Due to collecting multiple observations per participant, relatively small sample sizes are required.

In the main phase, we plan to recruit 140 patients and expect approximately 25-30% of carers of them participate as well, this gives us an estimate of 190 participants. We will record multiple observations from each participant (18 data lines per participant—3 alternatives X 6 choice sets). This is within the middle range of samples size of DCEs reviewed, including six in palliative care[2-7] which had 80 to 350 participants. Thus our study will be consistent with usual best practice.

Design of choice questions

Combination of all attributes produced 864 (3 X 2 X 4 X 3 X 3 X 4) profiles. Pairs of profiles were to be presented to respondents. Due to practicality and cognitive burden, we reduced the number of pairs with a D-optimal design strategy,[26] after removing implausible combinations, resulting in 18 sets of choices (36 profiles). To further reduce the number of choices presented, we used block design by randomisation. Each respondent faced six choices (see Supplementary Figure S1 for an example choice) and one warm-up at the beginning which was not used in the analysis. We included a third alternative (*Neither*, in which case patients do what they do to manage and treat their breathlessness) in each choice, which represents the current treatment or management of breathlessness and prevents us from overestimating preferences or acceptance.

Administration of discrete choice experiment questionnaire

DCE questions were administered with background, contextual information and task instructions. We gave a written vignette to respondents and read it aloud to make respondents familiar with the settings and services that BSS was suggesting. We also explain the common and/or different aspects of the BSS from the pulmonary rehabilitation service provided in NHS. We described attributes again prior to starting. Then,

Yi D, *et al. Thorax* 2023; 78:489–495. doi: 10.1136/thoraxjnl-2021-218251

Supplementary information to Yi et al.

there was a warm-up exercise where we asked the participants to describe their current health care service to manage breathlessness in terms of attributes (this tested their understanding of the task, attributes and levels as well as how well they knew the characteristics of the service they were on) and a warm-up example choice set with annotations. We used icons for each level in attributes, unveiling and reading aloud attributes one by one to ensure participants consider each and every one of them when making choices. The vocabulary was user-friendly, checked and improved in pre-pilot and pilot stages, asking participants to explain why they chose the option for each task to detect heuristics and need for improvements in the choice tasks.

Supplementary information to Yi et al.

Table S1. Participating organisations and recruited participants

Organisation	Participant (n)		
	Patient	Carer	Total
King’s College Hospital NHS Foundation Trust	61	27	88
Guy's and St Thomas' NHS Foundation Trust	26	15	41
Lewisham and Greenwich NHS Trust	6	3	9
South Tyneside NHS Foundation Trust	29	12	41
Derby Hospitals NHS Foundation Trust	13	0	13
Weston Area Health NHS Trust	8	3	11
Sherwood Forest Hospitals NHS Foundation Trust	27	2	29
Epsom and St Helier University Hospital NHS Trust	6	3	9
Cambridgeshire and Peterborough NHS Foundation Trust	14	3	17

Supplementary information to Yi et al.

Table S2. Parameters in the Markov model in deterministic analysis

Variables			Parameter
Age			75
Uptake probability	Best scenario (BSS I*)	Man	0.85
		Woman	0.87
	Worst scenario (BSS II*)	Man	0.33
		Woman	0.55
Health and social care costs	Usual care	Initial status	£3,709
		For 12 weeks	£2,816
	BSS plus Usual care	Initial status	£2,911
		For 12 weeks	£2,844
	BSS with lasting effects	Initial status	£2,911
		For 24 weeks	£2,844
Intervention costs			£357.94
QALYs	Usual care	Initial status	0.35
		For 12 weeks	0.34
	BSS plus Usual care	Initial status	0.35
		For 12 weeks	0.44
	BSS with lasting effects	Initial status	0.35
		For 24 weeks	0.44

Notes: **BSS I** involves the consultations with specialist at outpatient clinic, reviewing both medicinal & non-medicinal treatments, home visits by therapists and support from a social worker. Better mobility and independence at home and outside home, and more social activities are anticipated. Fewer hospital admissions are expected, and patients need to wait 2 weeks to get the first appointment. **BSS II** offers two consultations with GPs or nurses at GP surgeries, reviewing medicinal treatments. There is no additional support provided. Better mobility and independence at home and fewer visits to GP surgeries are anticipated. Waiting time for the first appointment is 8 weeks. Probabilities of taking part in the BSS is derived from the discrete choice experiments data analysis by gender.

Yi D, *et al. Thorax* 2023; 78:489–495. doi: 10.1136/thoraxjnl-2021-218251

Supplementary information to Yi et al.

Table S3. All-cause mortality rate and respiratory mortality rate by age and sex used in the Markov model

	All cause		COPD	
Age	Men	Women	Men	Women
65	0.012319	0.00791		
66	0.013587	0.008923	0.0145	0.0094
67	0.014706	0.009654	0.0145	0.0094
68	0.01573	0.010405	0.0145	0.0094
69	0.017378	0.011582	0.0145	0.0094
70	0.019016	0.012751	0.0227	0.0151
71	0.021536	0.014342	0.0227	0.0151
72	0.023826	0.016018	0.0227	0.0151
73	0.026358	0.018096	0.0227	0.0151
74	0.030029	0.019863	0.0227	0.0151
75	0.033498	0.022485	0.0398	0.0277
76	0.037101	0.025789	0.0398	0.0277
77	0.04067	0.027839	0.0398	0.0277
78	0.045081	0.03189	0.0398	0.0277
79	0.050449	0.035229	0.0398	0.0277
80	0.05679	0.040488	0.0694	0.0506
81	0.063335	0.045637	0.0694	0.0506
82	0.071779	0.05242	0.0694	0.0506
83	0.081743	0.060183	0.0694	0.0506
84	0.09171	0.068991	0.0694	0.0506
85	0.103257	0.077875	0.1241	0.0975
86	0.115855	0.089264	0.1241	0.0975
87	0.130367	0.101338	0.1241	0.0975
88	0.147147	0.116361	0.1241	0.0975
89	0.163395	0.131781	0.1241	0.0975

Source: Office for National Statistics, Top 10 causes of death by sex and age, England and Wales 1915-2015.
NHS Digital, Compendium of population health indicators, Mortality from bronchitis, emphysema and other COPD (ICD-10 J40 - J44 equivalent to ICD-9 490 - 492, 496), March 2019, Office for National Statistics deaths registered in England and Wales and mid-year population estimates

Yi D, *et al. Thorax* 2023; 78:489–495. doi: 10.1136/thoraxjnl-2021-218251

Supplementary information to Yi et al.

Table S4. Distribution of levels within attributes among choice questions chosen by participants

Attribute	Level	Choice (n)
Place of consultation	(Neither option)	213
	Home with GP or nurse	473
	GP surgery with GP or nurse	356
	Outpatient clinic with consultant	493
Treatment review	(Neither option)	213
	Non-medicinal	569
	Non-medicinal and medicinal	753
Additional support	(Neither option)	213
	None	267
	Physiotherapist and/or occupational therapist	365
	Social worker	301
	Therapist and social worker	389
Expectation for breathlessness	(Neither option)	213
	More mobile at home	415
	More mobile at home and outsides	460
	More mobile & social activities	447
Expectation for health service use	(Neither option)	213
	Fewer visit to GP clinic	424
	Fewer visit to A&E	423
	Fewer admission to hospital inpatient	475
Waiting time (weeks)	(Neither option)	213
	1 weeks	405
	2 weeks	329
	4 weeks	323
	8 weeks	265

Note: 213 choice questions out of 1,535 were answered to choose *Neither* option.

Supplementary information to Yi et al.

Table S5. Incremental cost effectiveness ratio of BSS for 5 years (unit: £)

	75 year old man			75 year old woman		
	No BSS	BSS offered	BSS with lasting effect	No BSS	BSS offered	BSS with lasting effect
	BSS I (P=0.85)			***BSS I*** (P=0.87)		
Costs (£)	57,281	56,618	52,195	58,989	58,240	53,270
	(27,540, 87,022)	(27,898, 85,338)	(28,011, 76,379)	(33,752, 84,226)	(33,889, 82,591)	(32,929, 73,611)
QALYs	1.325	1.338	1.338	1.363	1.376	1.367
	(0.659, 1.992)	(0.672, 2.005)	(0.790, 1.887)	(0.794, 1.931)	(0.808, 1.944)	(0.896, 1.837)
Δ costs (£)		-663	-5,086		-749	-5,719
		(-1,076, -250)	(-5,469, -4,703)		(-1,100, -398)	(-6,043, 5,395)
Δ QALYs		0.013	0.013		0.013	0.004
		(0.004, 0.022)	(0.004, 0.022)		(0.005, 0.021)	(-0.003, 0.011)
ICER		-50,789	-389,776		-56,242	-1,454,683
	BSS II (P=0.33)			***BSS II*** (P=0.55)		
Costs (£)	57,226	56,776	55,234	59,028	58,406	55,246
	(27,485, 86,967)	(28,046, 85,506)	(27,765, 82,703)	(33,791, 84,265)	(34,051, 82,761)	(32,873, 77,617)
QALYs	1.324	1.330	1.331	1.363	1.373	1.365
	(0.657, 1.990)	(0.664, 1.997)	(0.704, 1.958)	(0.795, 1.932)	(0.804, 1.941)	(0.856, 1.875)
Δ costs (£)		-450	-1,992		-622	-3,782
		(-864, -36)	(-2,397, -1,587)		(-973, -271)	(-4,119, -3,445)
Δ QALYs		0.006	0.007		0.009	0.002
		(-0.003, 0.016)	(-0.002, 0.016)		(0.001, 0.017)	(-0.006, 0.009)
ICER		-70,686	-274,607		-67,599	-2,200,392

Yi D, *et al. Thorax* 2023; 78:489–495. doi: 10.1136/thoraxjnl-2021-218251

Supplementary information to Yi et al.

Notes: 95% confidence intervals are in the parentheses. **BSS I** involves the consultations with specialist at outpatient clinic, reviewing both medicinal & non-medicinal treatments, home visits by therapists and support from a social worker. Better mobility and independence at home and outside home, and more social activities are anticipated. Fewer hospital admissions are expected, and patients need to wait 2 weeks to get the first appointment. **BSS II** offers two consultations with GPs or nurses at GP surgeries, reviewing medicinal treatments. There is no additional support provided. Better mobility and independence at home and fewer visits to GP surgeries are anticipated. Waiting time for the first appointment is 8 weeks. Probabilities of taking part in the BSS is derived from the discrete choice experiments data analysis by gender. Costs are in 2014 UK sterling pounds.

Supplementary information to Yi et al.

Table S6. Preferences for attributes and levels of Breathlessness Service: subgroup analysis by patient and carer

		**All**			**Patient**			**Carer**		
		β	95% CI		β	95% CI		β	95% CI	
Constant	BSS	1.519	1.310	1.729	1.562	1.314	1.809	1.425	1.021	1.828
Place of consultation	Home visit	0.145	0.056	0.235	0.113	0.009	0.217	0.248	0.067	0.429
	GP Surgery	-0.301	-0.394	-0.207	-0.305	-0.413	-0.196	-0.282	-0.473	-0.090
	Outpatient clinic	0.155	0.064	0.247	0.192	0.087	0.297	0.033	-0.156	0.223
Review	Non medicinal review	-0.147	-0.208	-0.086	-0.118	-0.189	-0.047	-0.246	-0.371	-0.121
	Both reviews	0.147	0.086	0.208	0.118	0.047	0.189	0.246	0.121	0.371
Additional support	No additional support	-0.231	-0.353	-0.110	-0.174	-0.314	-0.034	-0.403	-0.654	-0.153
	Therapists	0.197	0.080	0.314	0.240	0.105	0.376	0.088	-0.145	0.321
	Social worker	-0.154	-0.272	-0.035	-0.163	-0.300	-0.025	-0.152	-0.394	0.091
	Therapists and social worker	0.188	0.069	0.307	0.096	-0.043	0.236	0.467	0.228	0.706
Expectation 1	Mobile at home	-0.124	-0.216	-0.032	-0.117	-0.223	-0.011	-0.159	-0.350	0.032
	Mobile at home and outside	0.044	-0.047	0.135	0.026	-0.079	0.132	0.101	-0.084	0.287
	Mobile + Social activities	0.080	-0.017	0.177	0.091	-0.022	0.203	0.058	-0.140	0.256
Expectation 2	Avoid GP visit	-0.076	-0.168	0.016	-0.044	-0.149	0.062	-0.150	-0.342	0.042
	Avoid A&E	0.019	-0.075	0.114	0.010	-0.100	0.120	0.026	-0.164	0.216
	Avoid admission	0.056	-0.038	0.151	0.033	-0.077	0.143	0.124	-0.067	0.314
Time	Time for the first appointment	-0.170	-0.232	-0.109	-0.155	-0.226	-0.083	-0.226	-0.351	-0.100
N		4,605			2,640			1,965		
Loglikelihood		-1462.7443			-854.45591			-603.99594		

Notes: There was no difference between London and non-London where the null hypothesis of no difference was not rejected (LR X^2=21.77, p<0.04).

Supplementary information to Yi et al.

Table S7. Preferences for attributes and levels of Breathlessness Service: subgroup analysis by sex of patient

		**All**			**Woman patient**			**Man patient**		
		β	95% CI		β	95% CI		β	95% CI	
Constant	BSS	1.519	1.310	1.729	1.598	1.153	2.042	1.553	1.252	1.855
Place of consultation	Home visit	0.145	0.056	0.235	0.195	0.019	0.371	0.084	-0.046	0.213
	GP Surgery	-0.301	-0.394	-0.207	-0.234	-0.415	-0.053	-0.348	-0.486	-0.211
	Outpatient clinic	0.155	0.064	0.247	0.039	-0.144	0.222	0.265	0.135	0.395
Review	Non medicinal review	-0.147	-0.208	-0.086	-0.127	-0.245	-0.008	-0.113	-0.202	-0.023
	Both reviews	0.147	0.086	0.208	0.127	0.008	0.245	0.113	0.023	0.202
Additional support	No additional support	-0.231	-0.353	-0.110	-0.221	-0.457	0.015	-0.155	-0.331	0.020
	Therapists	0.197	0.080	0.314	0.349	0.123	0.575	0.201	0.028	0.373
	Social worker	-0.154	-0.272	-0.035	-0.389	-0.631	-0.147	-0.055	-0.224	0.115
	Therapists and social worker	0.188	0.069	0.307	0.261	0.027	0.494	0.009	-0.167	0.186
Expectation 1	Mobile at home	-0.124	-0.216	-0.032	-0.020	-0.200	0.160	-0.166	-0.300	-0.033
	Mobile at home and outside	0.044	-0.047	0.135	0.006	-0.172	0.184	0.036	-0.097	0.170
	Mobile+Soial activities	0.080	-0.017	0.177	0.014	-0.180	0.207	0.130	-0.009	0.269
Expectation 2	Avoid GP visit	-0.076	-0.168	0.016	-0.093	-0.277	0.091	-0.024	-0.155	0.107
	Avoid A&E	0.019	-0.075	0.114	0.048	-0.136	0.231	-0.020	-0.159	0.119
	Avoid admission	0.056	-0.038	0.151	0.045	-0.142	0.232	0.044	-0.094	0.182
Time	Time for the first appointment	-0.170	-0.232	-0.109	-0.087	-0.211	0.038	-0.194	-0.283	-0.105
N		4,605			1,167			2,217		
Loglikelihood		-1462.7443			-354.99113			-708.90951		

Notes: There was no difference between London and non-London where the null hypothesis of no difference was not rejected (LR X^2=797.69, p<0.001).

Supplementary information to Yi et al.

Table S8. Preferences for attributes and levels of Breathlessness Service: subgroup analysis by location (London and outside London)

		**All**			**London**			**Outside London**		
		β	95% CI		β	95% CI		β	95% CI	
Constant	BSS	1.519	1.310	1.729	1.449	1.179	1.719	1.655	1.319	1.991
Place of consultation	Home visit	0.145	0.056	0.235	0.113	-0.006	0.231	0.189	0.052	0.326
	GP Surgery	-0.301	-0.394	-0.207	-0.275	-0.399	-0.151	-0.339	-0.483	-0.195
	Outpatient clinic	0.155	0.064	0.247	0.162	0.042	0.283	0.150	0.009	0.291
Review	Non medicinal review	-0.147	-0.208	-0.086	-0.127	-0.208	-0.046	-0.174	-0.268	-0.080
	Both reviews	0.147	0.086	0.208	0.127	0.046	0.208	0.174	0.080	0.268
Additional support	No additional support	-0.231	-0.353	-0.110	-0.222	-0.382	-0.061	-0.242	-0.429	-0.056
	Therapists	0.197	0.080	0.314	0.164	0.010	0.319	0.242	0.062	0.422
	Social worker	-0.154	-0.272	-0.035	-0.157	-0.315	0.002	-0.154	-0.335	0.026
	Therapists and social worker	0.188	0.069	0.307	0.214	0.057	0.371	0.155	-0.029	0.339
Expectation 1	Mobile at home	-0.124	-0.216	-0.032	-0.115	-0.238	0.008	-0.137	-0.277	0.002
	Mobile at home and outside	0.044	-0.047	0.135	0.058	-0.063	0.178	0.023	-0.118	0.164
	Mobile+Soial activities	0.080	-0.017	0.177	0.058	-0.071	0.186	0.114	-0.035	0.264
Expectation 2	Avoid GP visit	-0.076	-0.168	0.016	-0.045	-0.168	0.077	-0.113	-0.252	0.027
	Avoid A&E	0.019	-0.075	0.114	0.013	-0.112	0.137	0.027	-0.119	0.173
	Avoid admission	0.056	-0.038	0.151	0.032	-0.093	0.158	0.085	-0.059	0.230
Time	Time for the first appointment	-0.170	-0.232	-0.109	-0.199	-0.281	-0.117	-0.140	-0.234	-0.046
N		4,605			2,640			1,965		
Loglikelihood		-1462.7443			-854.45591			-603.99594		

Notes: There was no difference between London and non-London where the null hypothesis of no difference was not rejected (LR X^2=8.58, p<0.73).

Supplementary information to Yi et al.

Figure S1. Example choice question presented to respondents

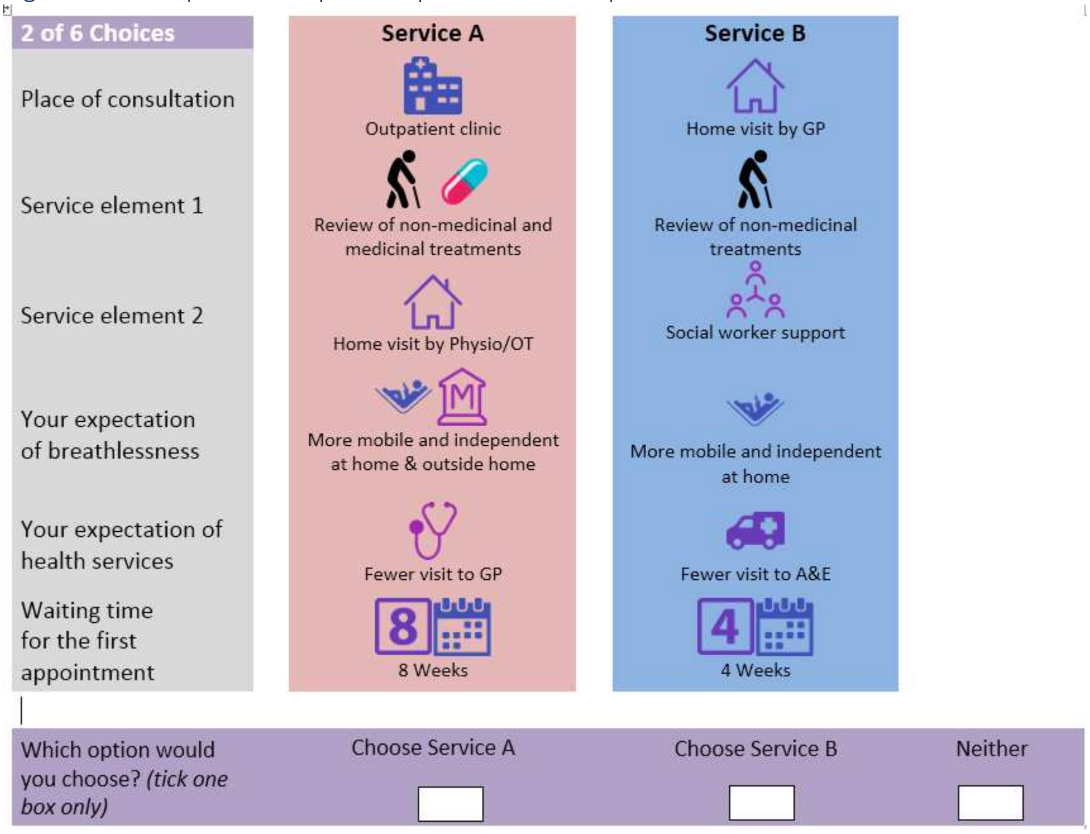

Supplementary information to Yi et al.

Figure S2. Decision tree for Markov model analysis of breathless support service

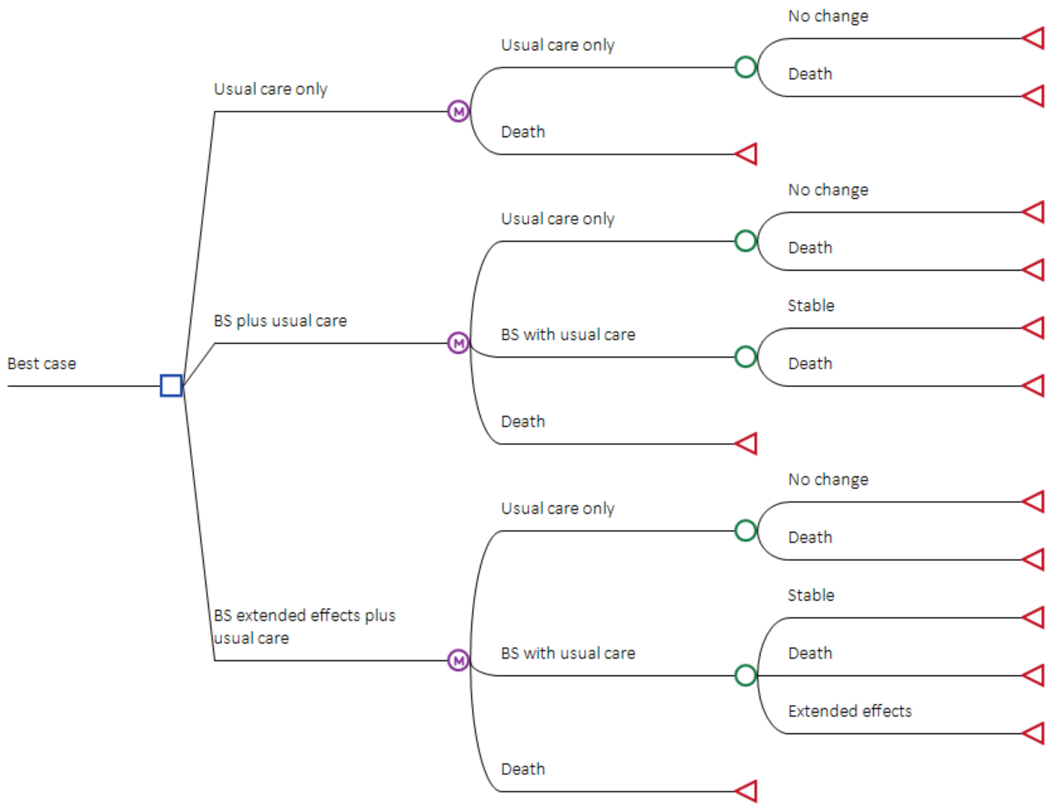

Notes: Probabilities of taking Breathlessness Services as well as age- and sex- specific, all cause and respiratory mortality (see Table S3) was used in defining the transitional chances. Simulations with 10,000 replications were estimated to generate costs and outcomes per person for 5 years (20 cycles).

Supplementary information to Yi et al.

References

1. Carmen R. Wilson VanVoorhis BLM. Understanding Power and Rules of Thumb for Determining Sample Sizes. *Tutorials in Quantitative Methods for Psychology* 2007;3(2):43-50.
2. Davison SN, Kromm SK, Currie GR. Patient and health professional preferences for organ allocation and procurement, end-of-life care and organization of care for patients with chronic kidney disease using a discrete choice experiment. *Nephrol Dial Transplant* 2010;25(7):2334-41. doi: 10.1093/ndt/gfq072 [published Online First: 2010/03/09]
3. Concordance in preferences for end of life care between advanced cancer patients and their caregivers in Singapore: A discrete choice experiment. Society for Medical Decision Making (SMDM) Asia-Pacific Conference; 2014; Singapore.
4. Morton RL, Snelling P, Webster AC, et al. Dialysis modality preference of patients with CKD and family caregivers: a discrete-choice study. *Am J Kidney Dis* 2012;60(1):102-11. doi: 10.1053/j.ajkd.2011.12.030 [published Online First: 2012/03/16]
5. Douglas HR, Normand CE, Higginson IJ, et al. A new approach to eliciting patients' preferences for palliative day care: the choice experiment method. *J Pain Symptom Manage* 2005;29(5):435-45. doi: 10.1016/j.jpainsymman.2004.08.017 [published Online First: 2005/05/21]
6. Hall J, Kenny P, Hossain I, et al. Providing Informal Care in Terminal Illness: An Analysis of Preferences for Support Using a Discrete Choice Experiment. *Med Decis Making* 2013 doi: 10.1177/0272989x13500719 [published Online First: 2013/08/15]
7. Casarett D, Fishman J, O'Dwyer PJ, et al. How Should We Design Supportive Cancer Care? The Patient's Perspective. *Journal of Clinical Oncology* 2008;26(8):1296-301. doi: 10.1200/jco.2007.12.8371
